# Supplementary material for: Gallium-Telluride-Based Composite as Promising Lithium Storage Material
Source: Nanomaterials (Basel). 2022 Sep 27;12(19):3362. doi: 10.3390/nano12193362 (PMC9565750; doi:10.3390/nano12193362)
Supplement: Supplementary file 1 [file nanomaterials-12-03362-s001.zip › nanomaterials-1890025-supplementary.pdf]

nanomaterials

MDPI

SUPPORTING INFORMATION

Gallium telluride-based composite for the promising lithium storage material

Vo Pham Hoang Huy, Il Tae Kim * and Jaehyun Hur *

Department of Chemical and Biological Engineering, Gachon University, Seongnam, Gyeonggi 13120, Korea
* Correspondence: itkim@gachon.ac.kr (I.T.K.); jhhur@gachon.ac.kr (J.H.)

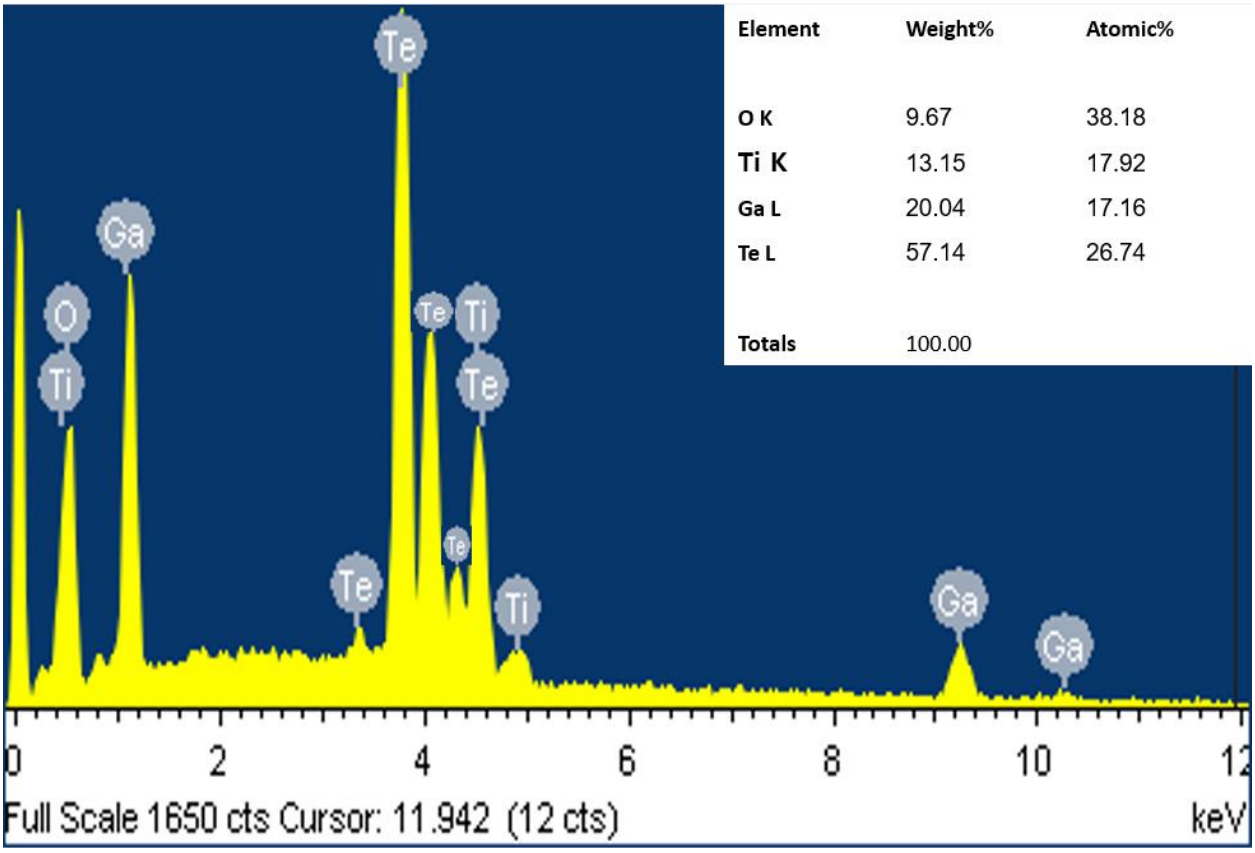

Figure S1. EDX spectrum of as-synthesized Ga_2Te_3-TiO_2.

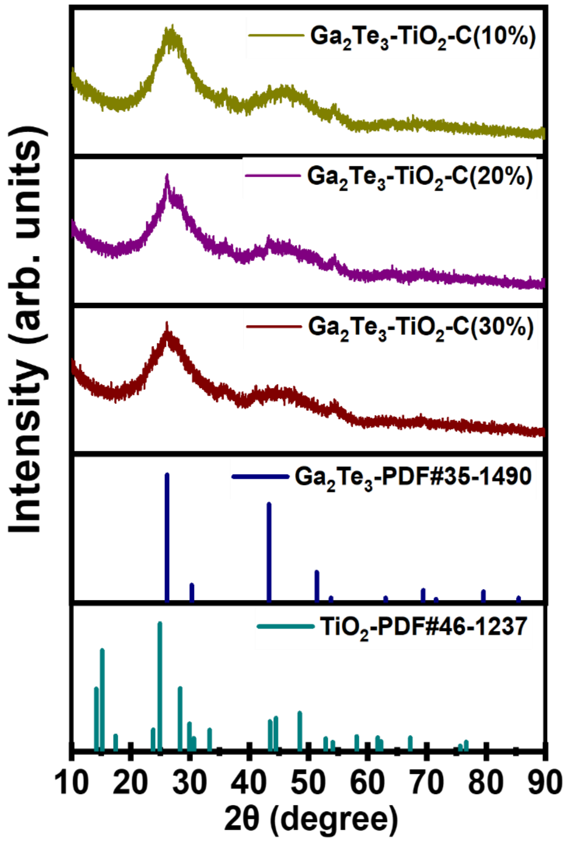

Figure S2. XRD pattern of Ga_2Te_3-TiO_2-C with different concentration of C.

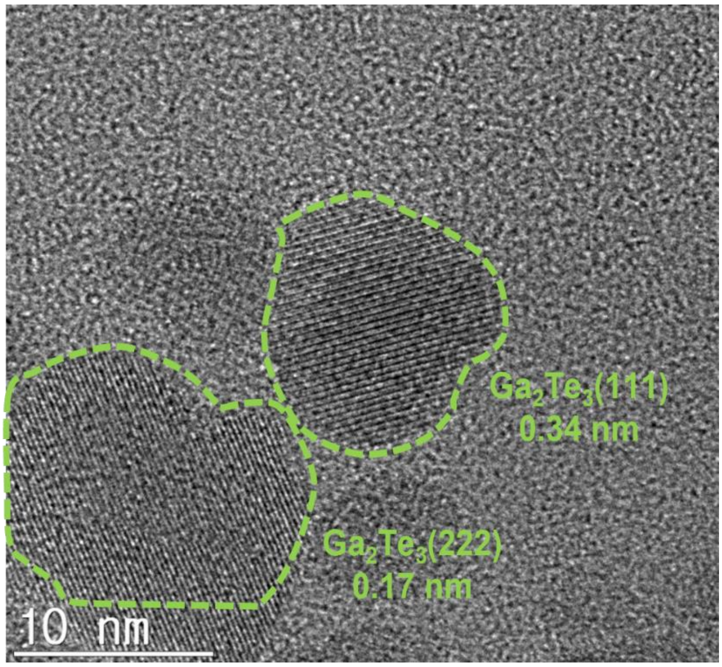

Figure S3. HRTEM image of Ga_2Te_3-TiO_2-C(10%).

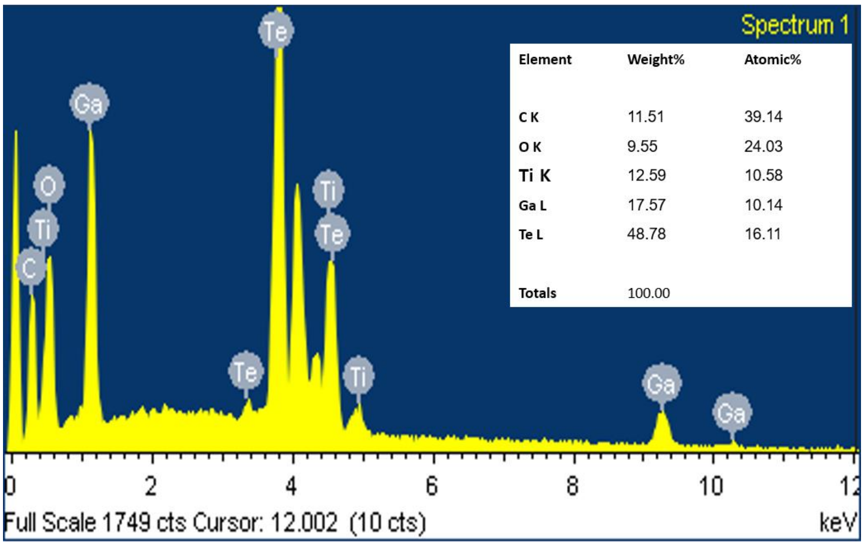

Figure S4. EDX spectrum of as-synthesized Ga_2Te_3-TiO_2-C(10%).

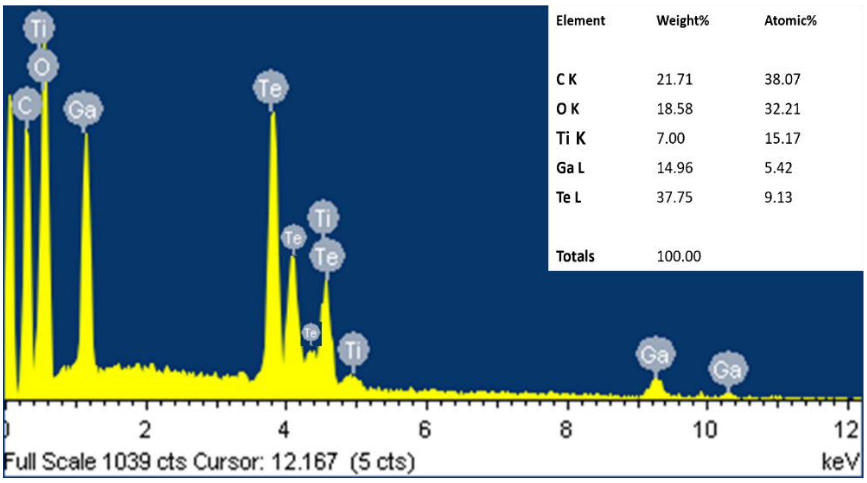

Figure S5. EDX spectrum of as-synthesized Ga_2Te_3-TiO_2-C(20%).

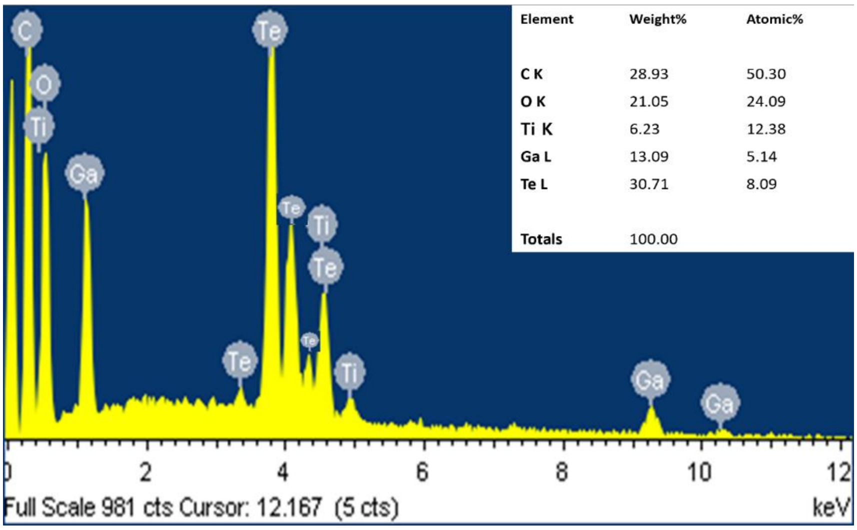

Figure S6. EDX spectrum of as-synthesized Ga_2Te_3-TiO_2-C(30%).

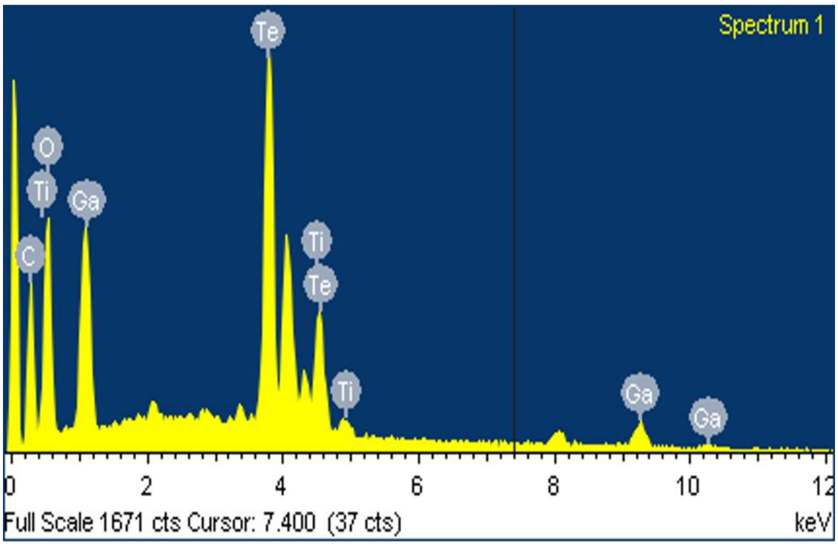

Figure S7. EDX spectrum of Ga_2Te_3-TiO_2-C(10%) anode.

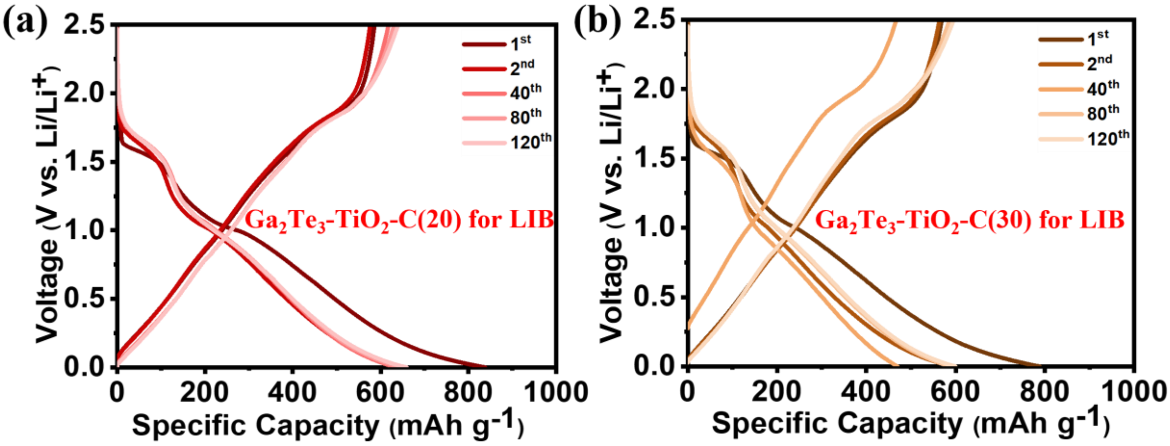

Figure S8. Galvanostatic discharge-charge profiles of (a) Ga_2Te_3-TiO_2-C(20%) and (b) Ga_2Te_3-TiO_2-C(30%).

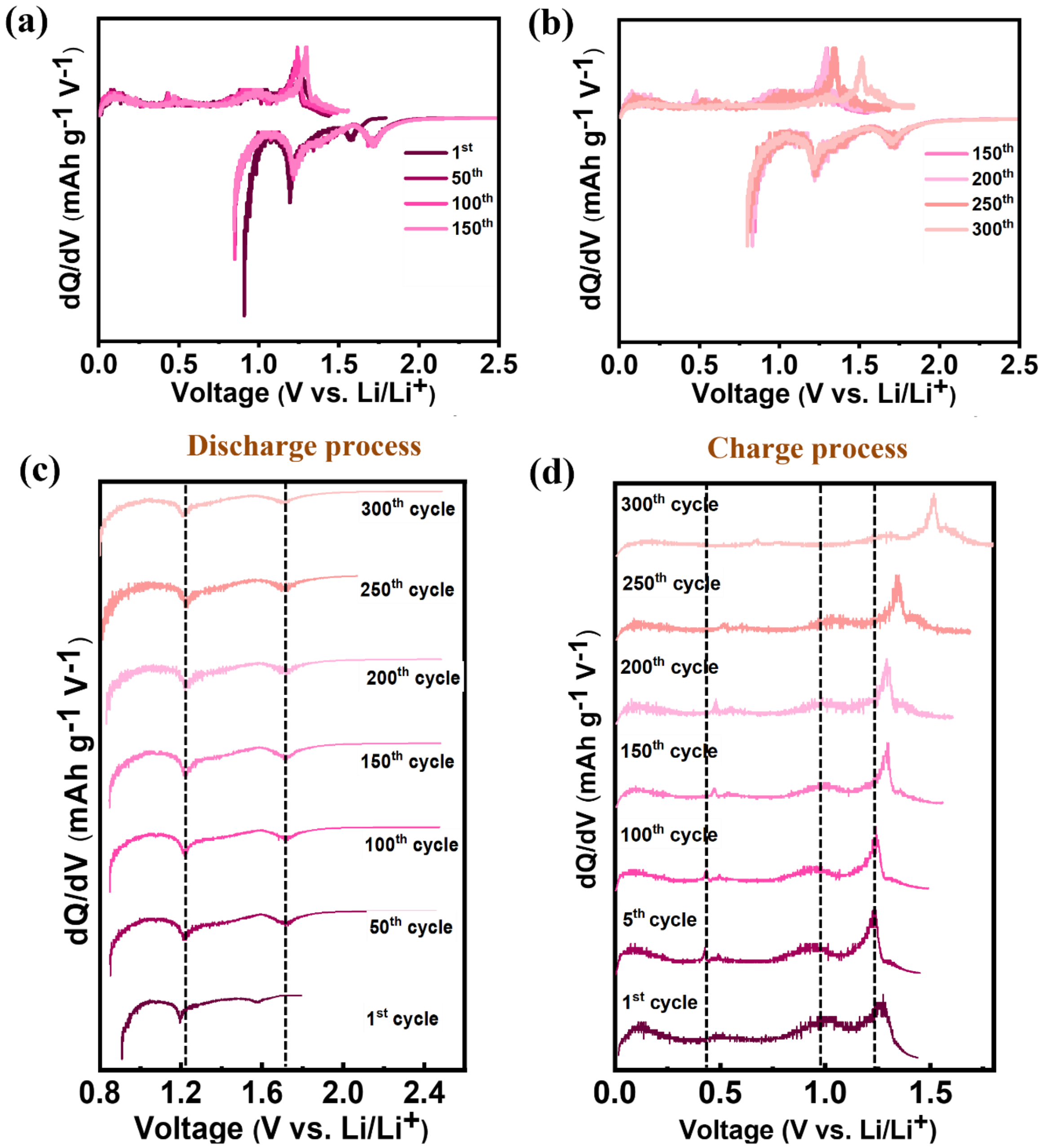

Figure S9. DCP profiles of Ga_2Te_3-TiO_2-C(10%) during 300 cycles measured at 100 mA g^{-1}: (a) 1–150 cycles and (b) 150–300 cycle. Enlarged view of (c) reduction and (d) oxidation peaks.

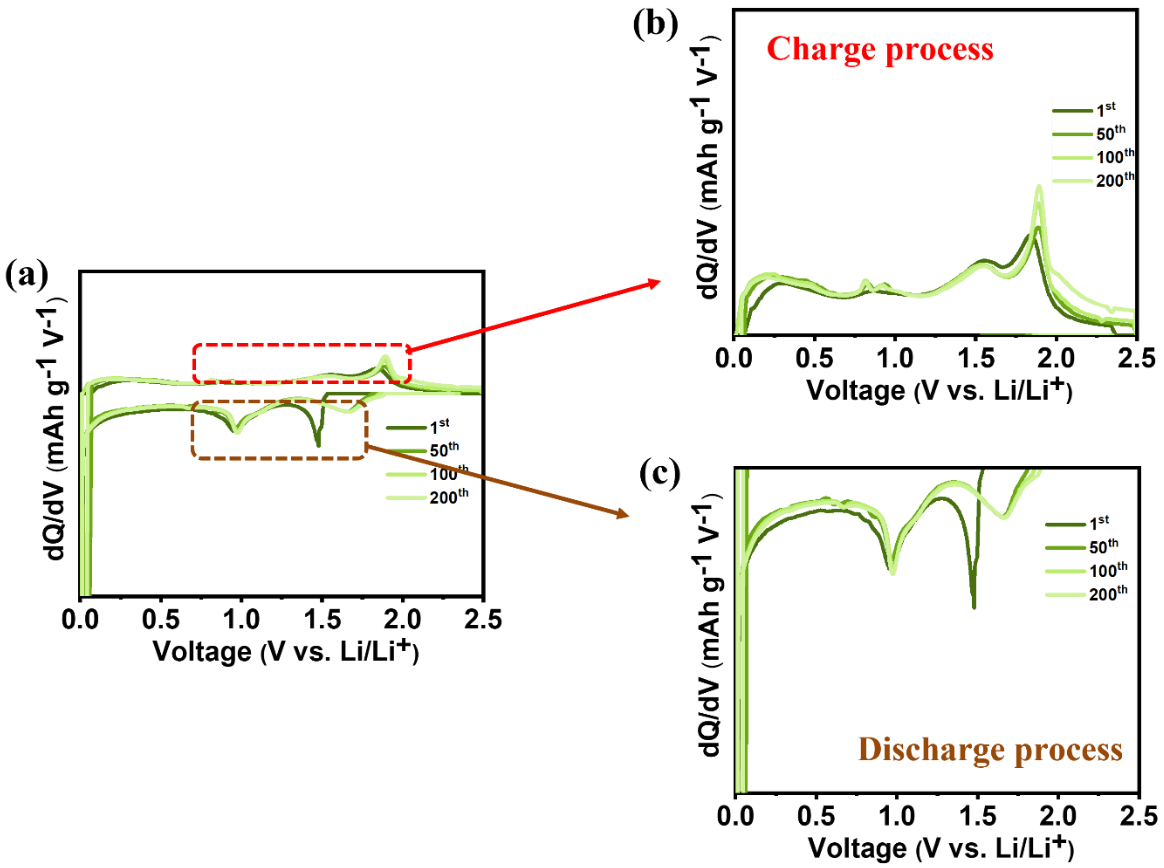

Figure S10. (a) DCP profiles of Ga_2Te_3-TiO_2-C(10%) during initial 200 cycles measured at 500 mA g^{-1}. Enlarged view of (b) oxidation and (c) reduction peaks.

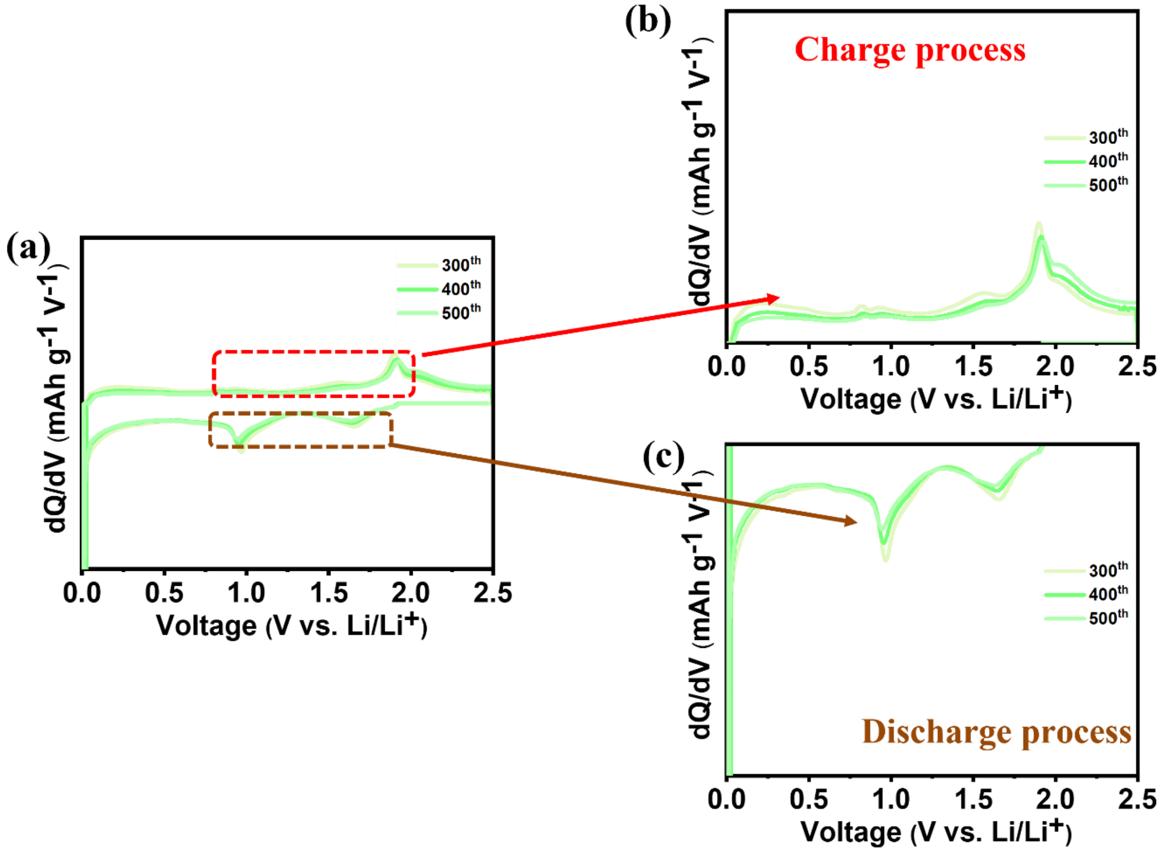

Figure S11. DCP profiles of Ga_2Te_3-TiO_2-C(10%) from 300 cycle to 500 cycles measured at 500 mA g^{-1}. Enlarged view of (b) oxidation and (c) reduction peaks.

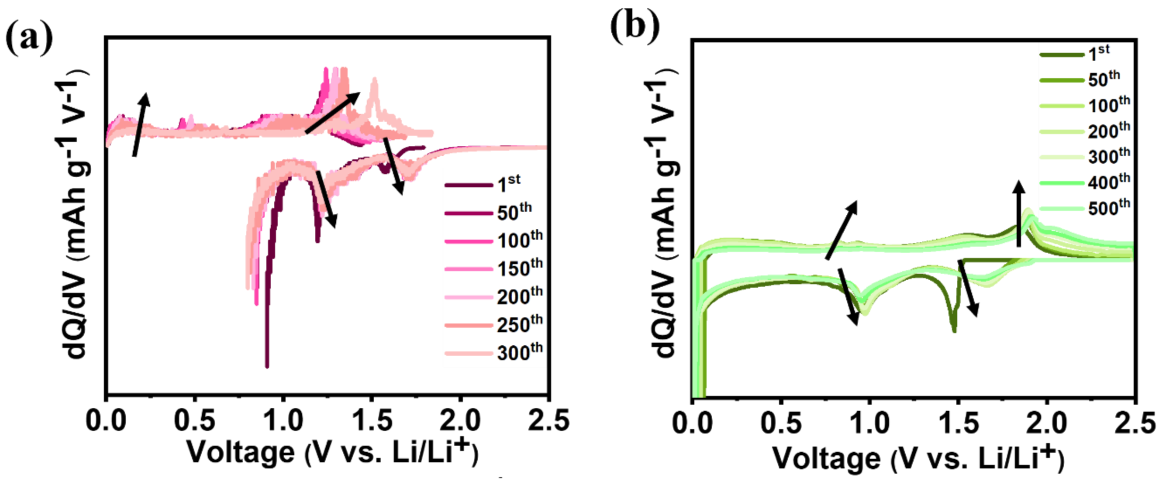

Figure S12. DCP profiles of Ga_2Te_3-TiO_2-C(10%) at current density (a) at 100 mA g^{-1} and during 300 cycles and (b) at 500 mA g^{-1} during 500 cycles.

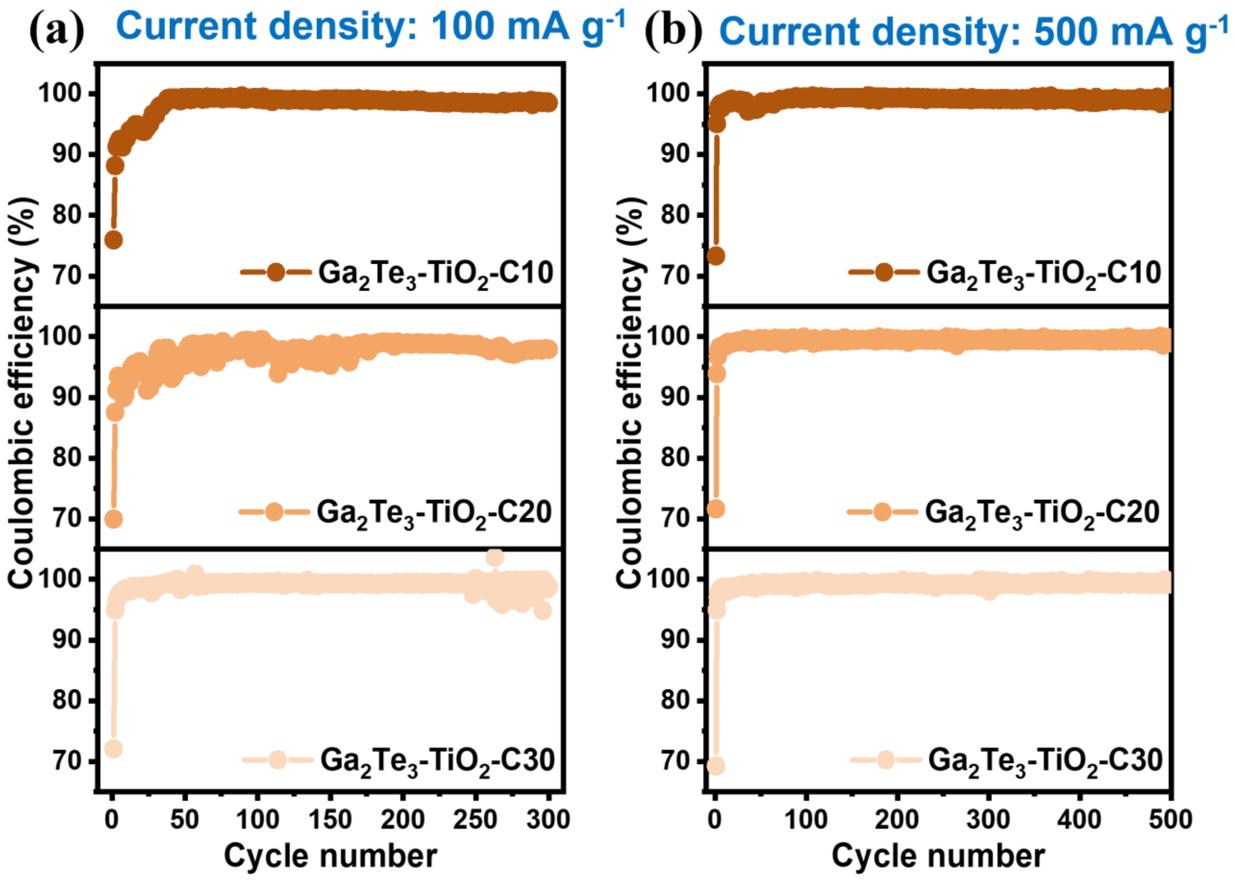

Figure S13. Coulombic efficiency of Ga_2Te_3-TiO_2 with different C content at current densities of (a) 100 and (b) 500 mA g^{-1}.

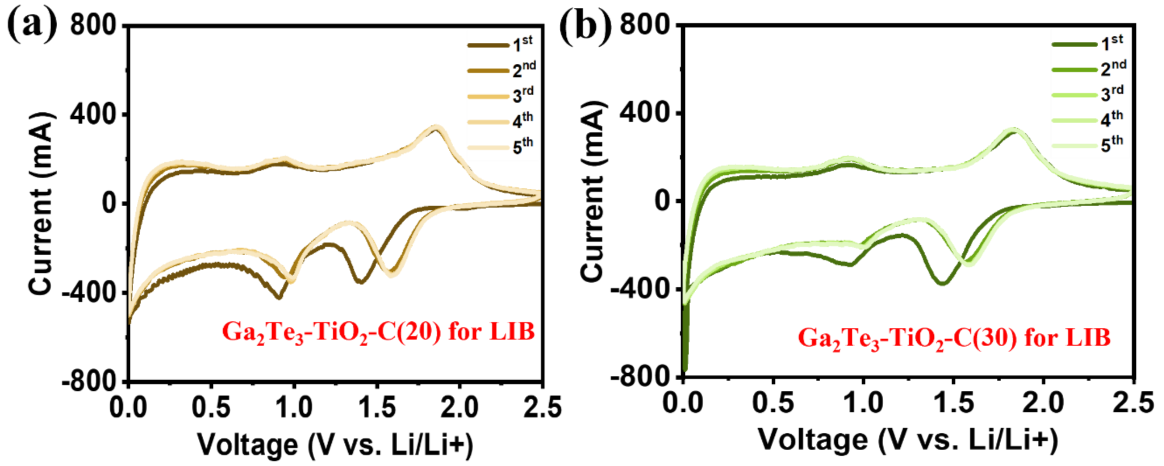

Figure S14. CV curves of (a) Ga_2Te_3-TiO_2-C(20%) and (b) Ga_2Te_3-TiO_2-C(30%) for LIBs.

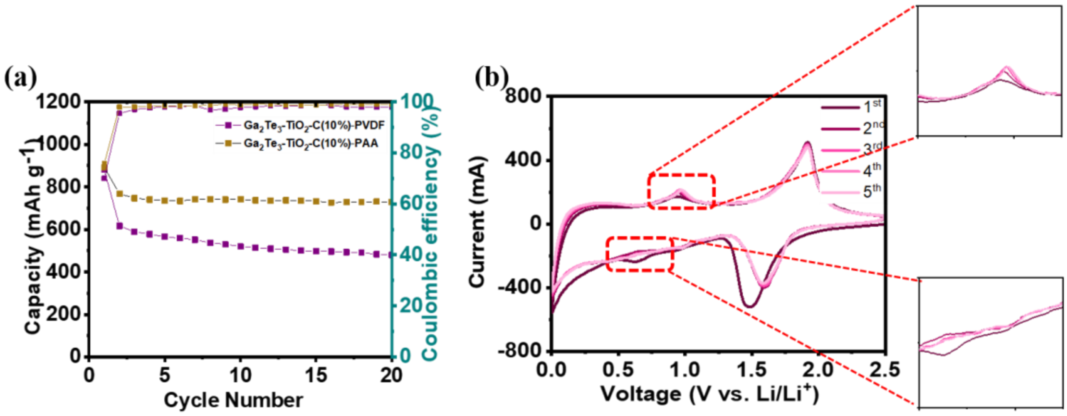

Figure S15. (a) Cycling performance of Ga_2Te_3-TiO_2-C (10%) with PAA and PVDF binder, (b) CV curves of Ga_2Te_3-TiO_2-C(10%) with PVDF binder.

Table S1. Calculation of capacity contribution of Ga_2Te_3, TiO_2 and C in the Ga_2Te_3-TiO_2-C(10%) composite in LIB.

	Ga_2Te_3	TiO_2	C
The mole of Li-ion participating reaction	10	1	0.17
Molecular weight (g mol^{-1})	522.24	79.9	12
Calculated theoretical capacity (mAh g^{-1})	~513	~336	~380
Actual weight faction in the Ga_2Te_3-TiO_2-C composite.	0.66	0.22	0.11
Contributed capacity (mAh g^{-1})	339	74	42
Capacity contribution (%)	75	16	9

Table S2. Calculation of capacity contribution of Ga_2Te_3, TiO_2 and C in the Ga_2Te_3-TiO_2-C(20%) composite in LIB.

	Ga_2Te_3	TiO_2	C
The mole of Li-ion participating reaction	10	1	0.17
Molecular weight (g mol^{-1})	522.24	79.9	12
Calculated theoretical capacity (mAh g^{-1})	~513	~336	~380
Actual weight faction in the Ga_2Te_3-TiO_2-C composite.	0.53	0.26	0.22
Contributed capacity (mAh g^{-1})	272	87	84
Capacity contribution (%)	61	20	19

Table S3. Calculation of capacity contribution of Ga_2Te_3, TiO_2 and C in the Ga_2Te_3-TiO_2-C(30%) composite in LIB.

	**Ga_2Te_3**	**TiO_2**	**C**
The mole of Li-ion participating reaction	10	1	0.17
Molecular weight (g mol^{-1})	522.24	79.9	12
Calculated theoretical capacity (mAh g^{-1})	~513	~336	~380
Actual weight faction in the Ga_2Te_3-TiO_2-C composite.	0.44	0.27	0.29
Contributed capacity (mAh g^{-1})	226	91	110
Capacity contribution (%)	53	21	26

Table S4. Calculation of theoretical capacity of Ga_2Te_3-TiO_2-C(10%) and Ga_2Te_3-TiO_2 in LIB.

**Anode materials**	**Ga_2Te_3-TiO_2-C(10%)**			**Ga_2Te_3-TiO_2**		
Component	Ga_2Te_3	TiO_2	C	Ga_2Te_3	TiO_2	C
Theoretical weight faction	0.66	0.22	0.11	0.80	0.20	-
Theoretical capacity (mAh g^{-1})	~513	~336	~380	~513	~336	~380
Contributed theoretical capacity (mAh g^{-1})	~339	~74	~42	~410	~67	-
Total theoretical capacity (mAh g^{-1})	~455			~477		

Table S5. Coulombic efficiency variation of Ga_2Te_3-TiO_2-C (10%) at various cycle numbers measured at 100 mA g^{-1} for LIB.

**Cycle number**	**Coulombic efficiency (%)**
1	75.93
10	92.64
20	94.57
50	99.34
100	99.11
150	99.13
200	98.92
250	98.62
300	98.54

Table S6. Coulombic efficiency variation of Ga_2Te_3-TiO_2-C(10%) at various cycle numbers measured at 500 mA g^{-1} for LIB.

Cycle number	Coulombic efficiency (%)
1	73.29
10	98.56
20	98.62
50	98.33
100	99.39
200	99.30
300	99.15
400	98.65
500	98.94

Table S7. Coulombic efficiency of Ga_2Te_3-TiO_2-C at current density of 100 mA g^{-1} during initial 10 cycles for LIB.

Cycle number	Coulombic efficiency (%)		
	Ga_2Te_3-TiO_2-C (10%)	Ga_2Te_3-TiO_2-C (20%)	Ga_2Te_3-TiO_2-C (30%)
1st	75.94	69.95	72.07
2nd	88.19	87.55	94.97
3rd	91.29	91.19	96.52
4th	91.99	93.49	97.24
5th	92.48	93.18	97.55
6th	92.55	93.38	97.91
7th	93.24	93.24	97.78
8th	95.99	89.98	98.27
9th	96.84	90.53	97.89
10th	98.64	93.62	98.48

Table S8. Coulombic efficiency of Ga_2Te_3-TiO_2-C at current density of 500 mA g^{-1} during initial 10 cycles for LIB.

Cycle number	Coulombic efficiency (%)		
	Ga_2Te_3-TiO_2-C (10%)	Ga_2Te_3-TiO_2-C (20%)	Ga_2Te_3-TiO_2-C (30%)
1st	73.29	71.63	69.26
2nd	95.11	93.92	94.98
3rd	97.31	96.74	97.05
4th	97.89	97.89	97.76
5th	98.16	98.29	98.19
6th	98.37	98.22	98.44
7th	97.68	98.31	98.53
8th	98.44	98.47	98.54
9th	98.42	98.42	98.67
10th	98.56	98.40	98.18

Table S9. Charge-transfer resistance (R_{ct}) of Ga_2Te_3-TiO_2-C for LIB.

	Ga_2Te_3-TiO_2-C (10%)	Ga_2Te_3-TiO_2-C (20%)	Ga_2Te_3-TiO_2-C (30%)
1 cycle	646.7 Ω	512.3 Ω	249.8 Ω
5 cycles	127.9 Ω	140.03 Ω	183.37 Ω
20 cycles	24.6 Ω	56.9 Ω	76.4 Ω
